# Computational prediction of human metabolic pathways from the complete human genome

**DOI:** 10.1186/gb-2004-6-1-r2

**Published:** 2004-12-22

**Authors:** Pedro Romero, Jonathan Wagg, Michelle L Green, Dale Kaiser, Markus Krummenacker, Peter D Karp

**Affiliations:** 1 Bioinformatics Research Group, SRI International, 333 Ravenswood Ave, Menlo Park, CA 94025, USA; 2 Department of Developmental Biology, Stanford University, Stanford, CA 94305, USA; 3 Current address: School of Informatics, Center for Computational Biology and Bioinformatics, Indiana University - Purdue University Indianapolis, 714 N Senate Ave, Indianapolis, IN 46202, USA

## Abstract

**Background:**

We present a computational pathway analysis of the human genome that assigns enzymes encoded therein to predicted metabolic pathways. Pathway assignments place genes in their larger biological context, and are a necessary first step toward quantitative modeling of metabolism.

**Results:**

Our analysis assigns 2,709 human enzymes to 896 bioreactions; 622 of the enzymes are assigned roles in 135 predicted metabolic pathways. The predicted pathways closely match the known nutritional requirements of humans. This analysis identifies probable omissions in the human genome annotation in the form of 203 pathway holes (missing enzymes within the predicted pathways). We have identified putative genes to fill 25 of these holes. The predicted human metabolic map is described by a Pathway/Genome Database called HumanCyc, which is available at . We describe the generation of HumanCyc, and present an analysis of the human metabolic map. For example, we compare the predicted human metabolic pathway complement to the pathways of *Escherichia coli *and *Arabidopsis thaliana *and identify 35 pathways that are shared among all three organisms.

**Conclusions:**

Our analysis elucidates a significant portion of the human metabolic map, and also indicates probable unidentified genes in the genome. HumanCyc provides a genome-based view of human nutrition that associates the essential dietary requirements of humans with a set of metabolic pathways whose existence is supported by the human genome. The database places many human genes in a pathway context, thereby facilitating analysis of gene expression, proteomics, and metabolomics datasets through a publicly available online tool called the Omics Viewer.

## Abstract 2

A computation pathway analysis of the human genome is presented that assigns enzymes encoded by the genome to predicted metabolic pathways. This analysis provides a genome-based view of human nutrition.

## Background

The human genome is a blueprint, but for what machinery? One approach to understanding the complex processes encoded by the human genome is to assign its enzyme products to biochemical pathways that define regulated sequences of biochemical transformations. Pathway and interaction assignments place genes in their larger biological context, and enable causal inferences about the likely effects of mutations, drug interventions and changes in gene regulation. They are a first step toward quantitative modeling of metabolism. Assignment of genes to pathways also permits a validation of the human genome annotation because patterns of pathway assignments spotlight likely false-positive and false-negative genome annotations. For example, false-negative assignments appear as pathway holes: missing enzymes within a pathway that are likely to be hiding in the genome.

SRI's Bioinformatics Research Group has developed a pathway-bioinformatics technology called a pathway/genome database (PGDB), which describes the genome, the proteome, the reactome and the metabolome of an organism. A PGDB describes the replicons of an organism (chromosome(s) or plasmid(s)), its genes, the product of each gene, the biochemical reaction(s), if any, catalyzed by each gene product, the substrates of each reaction, and the organization of those reactions into pathways. Pathway Tools is a reusable software environment for constructing and managing PGDBs [1]. It supports many operations on PGDBs including PGDB creation, querying and visualization, analysis, interactive editing, web publishing, and prediction of the metabolic-pathway complement of an organism.

The power of Pathway Tools is derived from both its database schema, and its software components. Both were originally developed for the EcoCyc project [2,3]. A PGDB can be thought of as a symbolic computational theory of a species' metabolic functions and genetic interactions [4], encoding knowledge in a manner suitable for computational analysis. Indeed, once an organism's genome and biochemical network are encoded within the schema of a PGDB, new possibilities for symbolic computational analysis arise, because many important semantic relationships are described in a computable fashion.

PathoLogic is one of the Pathway Tools software components. Its primary function is to generate a new PGDB from an organism's annotated genome. PathoLogic predicts the metabolic pathways of the organism, providing new global insights about its biochemistry, and generates reports that summarize the evidence for the presence of each predicted metabolic pathway. We used PathoLogic to generate HumanCyc, a PGDB for *Homo sapiens*, from the annotated human genome. The genome data used as input to PathoLogic combined data from the Ensembl database [5], the LocusLink database [6] and GenBank [7].

Our analysis assigns 2,709 human enzymes to 135 predicted metabolic pathways. It provides a genome-based view of human nutrition that associates the essential dietary requirements of humans that were previously derived mainly from animal and tissue extract studies to a set of metabolic pathways whose existence is derived from the human genome. The analysis also identifies probable omissions in the human-genome annotation in the form of pathway holes (missing enzymes within the predicted pathways); we have identified putative genes to fill some of those pathway holes. This paper describes the generation of HumanCyc, and presents an analysis of the human metabolic map. The computationally predicted pathways are consistent with known human dietary requirements. We compare the predicted human metabolic pathway complement to the pathways of *Escherichia coli *and *Arabidopsis thaliana *and identify 35 pathways that are shared among all three organisms, and therefore define an upper bound on a potential set of universally occurring metabolic pathways.

## Results

### Prediction of human metabolic pathways

We applied PathoLogic to the input files containing the *H. sapiens *annotated genome, as described in Materials and methods, generating HumanCyc.

**Figure 1:**
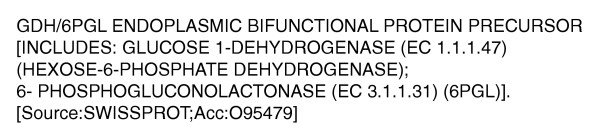
A typical description of a gene product's function in Ensembl. This example aims to communicate to the reader exactly what information was obtained from Ensembl; it shows multiple functions, synonyms and EC numbers, as well as a Swiss-Prot accession number, all in one line of text. A Perl script was developed to parse these descriptions and extract the relevant information.

Table 1 shows the results of PathoLogic's enzyme matching during the PGDB automated build. This computational matching process found more than 2,300 matches between gene products in the annotated genome and reactions in MetaCyc. Both the ambiguous matches (row 3 in Table 1) and the proteins labeled as 'probable enzymes' by PathoLogic (row 5) were examined manually; about half of them were manually matched to enzymes, as explained in Materials and methods. Sometimes one gene product is matched to more than one reaction, as happens with multifunctional enzymes (for example, the gene product shown in Figure 1 would be matched to two different reactions). So the number of matches is higher than the number of proteins matched. The 'Unmatched' row includes human proteins that are not enzymes.

Table 2 shows statistics from version 7.5 of HumanCyc (released in August 2003), after manual refinement of the PGDB was completed. The 2,742 enzyme genes in HumanCyc correspond to 9.5% of the human genome, and can be subdivided into 1,653 metabolic enzymes, plus 1,089 nonmetabolic enzymes (including enzymes whose substrates are macromolecules, such as protein kinases and DNA polymerases). Our best estimate of the total number of human metabolic enzymes is the sum of the 1,653 known enzymes plus the 203 pathway holes, for a total of approximately 6.5% of the human genome allocated to small-molecule metabolism (compared to 16% of the *E. coli *genome). Of the 1,653 metabolic enzymes, 622 are assigned to a pathway in HumanCyc, and the remainder are not assigned to any pathway; we expect that in the future some of the latter group of enzymes will be assigned to some known human pathways not yet in HumanCyc, and to some human pathways that remain to be discovered. Of the metabolic enzymes, 343 are multifunctional. The number of enzymes is less than the number of enzyme genes because, in many cases, the products of multiple genes are required to form one active enzyme complex.

**Figure 2:**
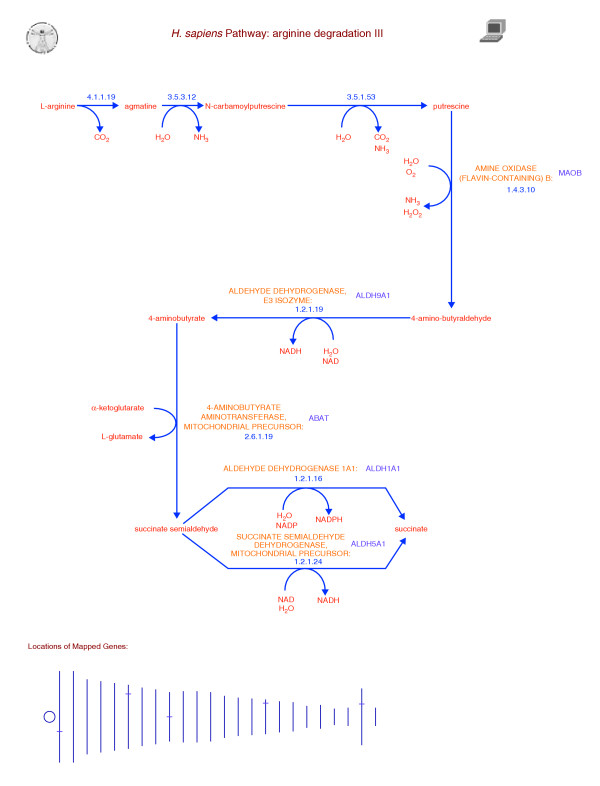
Predicted HumanCyc pathway for arginine degradation. The computer icon in the upper-right corner indicates this pathway was predicted computationally. Neither enzyme names nor gene names are drawn adjacent to the first three reactions of this pathway to indicate that these steps are pathway holes, meaning no enzyme has been identified for these steps in the human genome. The graphic at the bottom indicates the positions of genes within this pathways on the human chromosomes. Moving the mouse over a gene in the webpage for this diagram will identify the gene and the chromosome.

**Figure 3:**
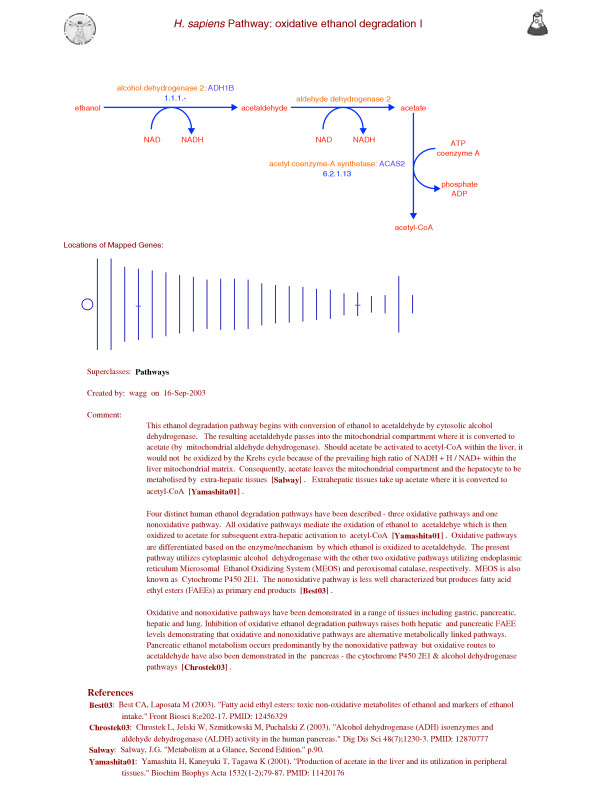
Curated HumanCyc pathway for oxidative ethanol degradation. This pathway was not predicted by PathoLogic, but was entered into HumanCyc as part of our subsequent literature curation effort. The flask icon in the upper-right corner indicates this pathway is supported by experimental evidence. The complete comment for this pathway is available at [38]

Table 3 shows all pathways present in HumanCyc, arranged according to the MetaCyc pathway taxonomy. Only the top two levels in the taxonomy are shown for the sake of brevity. The 135 metabolic pathways in HumanCyc is a lower bound on the total number of human metabolic pathways; this number excludes the 10 HumanCyc superpathways that are defined as linked clusters of pathways. The average length of HumanCyc pathways is 5.4 reaction steps. Example HumanCyc pathways are shown in Figures 2 and 3. All HumanCyc pathways can be accessed online from the HumanCyc Pathways page [8].

HumanCyc 7.5 contains 1,093 biochemical reactions, 896 of which have been assigned to one or more of the 2,709 enzymes in HumanCyc. There are more enzymes than reactions because of the existence of isozymes in the human genome. This leaves 203 reactions that have no assigned enzyme. These reactions correspond to the above-mentioned pathway holes for the HumanCyc pathways. Of the 896 reactions that have assigned enzymes, 428 have multiple isozymes assigned.

### Filling holes in HumanCyc pathways

The PathoLogic-based analysis of the annotated human genome inferred 135 metabolic pathways. A total of 203 pathway holes (missing enzymes) were present across 99 of these pathways; that is, 38 pathways were complete. Using our hole-filling algorithm [9], no candidate enzymes were found for 115 of the 203 pathway holes. For the remaining 88 pathway holes, candidates were obtained and evaluated. In 25 of these 88 cases putative enzymes were identified with sufficiently strong support that the enzyme and pathway annotations within HumanCyc have been updated to reflect these findings. See the HumanCyc release note history [10] for a list of these 25 hole fillers added to HumanCyc version 7.6.

The original annotations of the human proteins that were identified as candidate hole fillers fell into several classes: A description of each class is presented below, with examples included for some.

#### Open reading frames (ORFs) with no assigned function (6 candidates)

Putative enzymes were identified, for example, for the *N*-acetylneuraminate lyase (LocusLink ID 80896), aldose 1-epimerase (LocusLink ID 130589) and imidazolonepropionase (LocusLink ID 144193) reactions. In each of these cases, the function of the protein was previously unknown.

#### Proteins assigned a nonspecific function (7 candidates)

The pathway hole filler assigned an enzyme previously annotated with a general function. For example, 'amine oxidase (flavin-containing) B' (LocusLink ID 4129), was assigned to a more specific reaction, putrescine oxidase. A 'fatty acid synthase' (LocusLink ID 54995) was identified to fill the 3-oxoacyl-ACP synthase reaction.

#### Proteins assigned a single function but which our analysis indicates are multifunctional (9 candidates)

In these cases the program is postulating an additional function for a gene that already has an assigned function. The pathway hole filler identified the enoyl-CoA hydratase enzyme (LocusLink ID 1892) as a potential hole filler for the 3-hydroxybutyryl-CoA dehydratase reaction in the lysine degradation and tryptophan degradation pathways. The dihydrofolate synthase hole in formylTHF biosynthesis was filled by the enzyme (LocusLink ID 2356) catalyzing the folylpolyglutamate synthase reaction.

#### Proteins that may have been assigned an incorrect specific function

Although our analyses of other pathway/genome databases have revealed examples we consider to have been assigned an incorrect function in the original annotation, our analysis of the 25 HumanCyc pathway holes that we filled revealed no candidates in this category.

The pathway hole filler not only identifies candidate proteins for each pathway hole, but also determines the probability that each candidate has the desired function. Table 4 displays the homology-based features used by the pathway hole filler to compute this probability. The table shows three example reactions, each with two candidate enzymes and the data gathered for each. The columns in the table display the computed probability that the candidate has the desired function; the number of query sequences that hit the candidate (number of hits); the E-value for the best alignment between the candidate and a query sequence (best E-value); the average rank of the candidate in the lists of BLAST hits; and the average percentage of each query sequence that aligns with the candidate.

In the first example, 28 imidazolonepropionase sequences from other organisms were retrieved from Swiss-Prot and the Protein Information Resource (PIR). Using BLAST, each sequence was used to query the human genome for candidate enzymes. Protein A was found in all of the 28 lists of BLAST hits. From the numbers in the table, it is fairly obvious that protein A is more likely to catalyze the imidazolonepropionase reaction than is protein B. In the second example, given the best E-value (1e-110) it is again not surprising that the computed probability that protein C has *N*-acetylglucosamine-6-phosphate deacetylase activity approaches 1.0. In the last example, both proteins have excellent BLAST E-values; in fact, the E-value for protein F indicates a better match with the query sequences than the E-value for protein E. In this case, protein E is found in 19 lists of BLAST hits versus four for protein F, and on average aligns with a much larger fraction of each query sequence. When examined in more detail, we discover that the four query sequences that identified candidate F in their BLAST output are multifunctional proteins with both aldose-1-epimerase activity and UDP-glucose 4-epimerase activity. Protein F aligns with the amino-terminal region of each of the four query sequences, and has no detected similarity in the carboxy-terminal regions. The UDP-glucose 4-epimerase activity lies in the amino-terminal region of each multifunctional query protein.

### Nutritional analysis of the human metabolic network

Nutritional requirements and their genetic and biochemical basis are thought to have evolved principally in prokaryotes, over billions of years [11]. Specific nutritional challenges have driven the evolution of metabolic pathways and the functional capabilities mediated by them. Indeed, eukaryotic life acquired the basic building blocks of metabolism, that is, sets of genes encoding enzymes that mediate specific metabolic pathways, from prokaryotic ancestors. One may define a metabolic pathway as a conserved set of genes that endow an organism with specific nutritional/metabolic capabilities, for example, the ability to grow in the absence of phenylalanine because of the ability to synthesize phenylalanine.

Current knowledge of human nutrition based on metabolic pathways is derived from various sources. One is clinical observation of inherited human metabolic diseases and nutrient deficiency states. For some pathways, like oxidative phosphorylation and the TCA cycle, direct studies of human tissues, such as human muscle biopsies, have been made. Nuclear magnetic resonance (NMR) has been used directly on humans to study aspects of carbohydrate and energy metabolism. Stable isotopes have been used to trace human metabolism, from which inferences about nutrition have been made. Dietary studies have been made in experimental mammals such as rats and mice and metabolic pathways experimentally elucidated in model organisms.

Here we compare previously accepted human nutritional requirements with pathways derived from the human genome to evaluate their agreement. For example, biosynthetic pathways for essential human nutrients, that is, substances that must be provided in the diet such as the essential amino acids and vitamins, would not be expected to occur in the human genome.

Integration of human genome data with clinical, biochemical, physiological and other data obtained both directly from humans and indirectly from model organisms should, over time, lead to a deeper understanding of human metabolism and its nutritional implications in health and disease. When the genome sequences of individuals are available, it may be possible to address questions about the variation in optimal nutrition from person to person. Explicit identification of specific areas of inconsistency will serve to focus ongoing experimental efforts to elucidate the molecular basis of human nutrition and metabolism.

For all of the nine amino acids essential for humans, PathoLogic did not predict the presence of a corresponding biosynthetic pathway (see Table 5) [12]. And for all of the 11 nonessential amino acids, PathoLogic did predict the presence of a corresponding biosynthetic pathway. For 12 of 13 essential human vitamins, PathoLogic did not predict the presence of a corresponding metabolic pathway (note that PathoLogic could not have predicted such a pathway for six of those vitamins because MetaCyc does not contain such a pathway). PathoLogic did predict the presence of a pathway called 'pantothenate and coenzyme A biosynthesis pathway', which is not expected given that pantothenate is an essential human nutrient. However, examination of the predicted pathway reveals that no enzymes in the first part of the pathway (biosynthesis of pantothenate) are present; all enzymes are in the portion of the pathway that synthesizes coenzyme A from pantothenate. Thus, this false-positive prediction can be attributed to the fact that MetaCyc does not draw a boundary between what should probably be considered two distinct pathways. No hard-and-fast rules are generally accepted as to how to draw boundaries between metabolic pathways; therefore the PathoLogic method cannot produce objective and well accepted pathway boundaries (nor can any other known algorithm).

### Comparative analysis of the metabolic networks of human, *E. coli *and *Arabidopsis*

Table 6 indicates whether or not each HumanCyc pathway is present in the EcoCyc *E. coli *PGDB and in the AraCyc PGDB for *A. thaliana *[13]. More precisely, we say a pathway is shared among multiple PGDBs if the same MetaCyc pathway has been predicted to be present in each PGDB; that is, if the pathway has exactly the same set of reactions in the PGDBs (the unique identifier of the MetaCyc pathway is reused in any PGDB to which the pathway is copied). The comparison does not consider how many pathway holes are in the PGDBs, but relies on the PathoLogic prediction (plus subsequent manual review) that the pathway is present; that is, if PathoLogic determines that the pathway is present despite its holes, the comparison considers it to be present. Note that we do not count the presence of related pathway variants; that is, if organism A contains pathway P and organism B contains a variant of P, we do not score this case as a shared pathway. Some shared pathways will include pathway holes.

**Figure 4:**
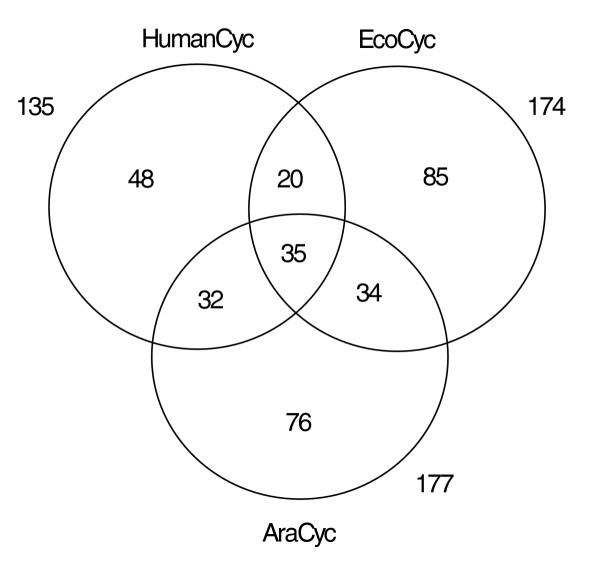
Numbers of pathways, including superpathways, shared by the three PGDBs HumanCyc (*H. sapiens*), EcoCyc (*E. coli*), and AraCyc (*A. thaliana*). The numbers outside the circles represent the total number of pathways in the corresponding PGDB. The numbers inside the intersecting areas represent the number of pathways that fall into each area. For example, there are 55 pathways in common between HumanCyc and EcoCyc (20 + 35). AraCyc contains 177 total pathways: 76 that are unique to *A. thaliana*, and 101 that are shared with other organisms.

Figure 4 shows how the three metabolic networks intersect by means of a Venn diagram, depicting each PGDB's pathway complement as a circle. The number within a given intersecting area denotes the number of pathways shared by the corresponding combination of PGDBs. For example, HumanCyc has 55 pathways in common with EcoCyc, as well as 67 with AraCyc, while EcoCyc and AraCyc share 69 pathways. Thirty-five pathways are common to all three databases, and are shown in Table 6. The 35 pathways include significant numbers of pathways from all the pathway classes (biosynthesis, catabolism and energy metabolism), and constitute a significant fraction of the pathway complements of both *E. coli *(20.1% of the 174 pathways in EcoCyc) and *H. sapiens *(25.7% of the 135 pathways in HumanCyc). Those 35 pathways therefore constitute a likely upper bound on the number of universally and exactly conserved metabolic pathways. It is an upper bound in the sense that as more organisms are considered, the list of universal pathways cannot grow larger.

We propose that the cofactor biosynthesis pathways shared among all three organisms have been conserved because first, they produce complex molecules that are not available from the environments of these organisms; second, these molecules are used as cofactors in so many reactions within the metabolic networks that the requirement for them is absolute; and third, no other pathway to accomplish the synthesis of that molecule has evolved. A study by Ouzounis and Karp surveyed global properties of the *E. coli *metabolic network, including the most frequently used substrates and cofactors [14]. Together, the two pyridine nucleotides NAD and NADP are the third most common substrate in the *E. coli *metabolic network (after water and ATP): removing the ability to synthesize NAD would disable so many reactions as to be insurmountable. Pyridoxal-5'-phosphate is the second most common cofactor (after Mg^2+^). Coenzyme A and acetyl-CoA together constitute the seventh most common substrate in *E. coli*, formylTHF constitutes the 23rd most common substrate, and thioredoxin and glutathione constitute the 40th and 41st most common substrates.

## Discussion

### Pathway variants

The level of metabolic pathway variation in the biosphere remains to be determined. Metabolic pathways have been experimentally elucidated in a small number of model prokaryotic and eukaryotic organisms. Despite the relatively small number of carefully studied organisms, significant pathway variation has been observed both between distinct organisms and within a given organism. For example, at least four variants of the 'glycolytic pathway' have been described [15]. Sets of variant pathways for glycolysis [16], leucine degradation [17], and NAD biosynthesis [18] can be viewed through the MetaCyc website. In MetaCyc, variant pathways are named with roman numerals; for example, 'NAD biosynthesis I' and 'NAD biosynthesis II'.

Metabolic pathways appear to have diverged in a manner analogous to the divergence of biological sequences. The demonstrated existence of pathway variants and ongoing uncertainty as to the full extent of such variation has significant implications for ongoing efforts to predict biochemical pathways from incomplete genomic data. First, it means that the precision with which we can infer pathways in one organism from another solely on the basis of genomic data remains to be determined, because when genomic evidence is found in organism O_k _for the presence of a pathway, P_j_, that was experimentally elucidated in an organism O_j_, this alone does not constitute conclusive evidence for the presence of P_j _in O_k_, since a variant of P_j _(P_k_) may be present in O_k _(note that any such P_k _variant may not even have been experimentally characterized). Second, for those pathways with known closely related variants (for example, two pathways differing by only a single step with one step differing from the other only at the level of co-reactants/products, such as one using NADP/NADPH the other using NAD/NADH as cosubstrates/products) it is often impossible to choose among these variants on the sole basis of genomic data because of the limited resolution of sequence analysis.

We have developed a general approach to representing the presence of pathway variants. MetaCyc pathways (approximately 500) have been grouped using a function-based classification system. Each grouping defines a 'pathway family', each member of which (a pathway variant) endows an organism with one or more specific functional capabilities, for example, the ability to grow in the absence of phenylalanine. Within a given pathway family, variants may be clustered into one or more subfamilies. Subfamilies are groups of pathways that show significant overlap/similarity in terms of individual reaction steps (reactants and products of each reaction); enzymatic activities that catalyze these steps; and genes encoding the enzymes that mediate these activities

The similarity between variants within a given subfamily suggests they evolved from a common ancestor pathway. For example, at least four variants of the glycolytic pathway have been described; all enable the conversion of glucose to pyruvate and show significant overlap/similarity in their component reactions. Other pathway variants have been observed that show little similarity to each other (for example, some of the amino-acid degradation pathways) and these are therefore believed to have evolved from distinct ancestor pathways. The existence of pathway subfamilies indicates that multiple pathways have coevolved to meet common nutritional/metabolic challenges.

For the purposes of this paper, genomic evidence for the presence of a specific biochemical pathway, P_1_, in humans is taken as evidence that P_1 _and/or other members of the pathway family to which P_1 _belongs are likely to be present in humans (including those not yet included in MetaCyc and/or experimentally elucidated). Indeed, PathoLogic sometimes inferred the presence of multiple variant pathways in humans. This occurred because when evidence was found in the genome for the presence of one member of a pathway family/subfamily, this evidence often also supported the presence of other members of this family/subfamily. In these cases, all inferred variants were included in HumanCyc. Of course, the specific members of a given pathway family actually present in humans may include one or more of those inferred from MetaCyc or other members of this pathway family not yet described in MetaCyc and/or not yet experimentally elucidated from any organism. It is attractive to think that multiple variant pathways might refer to metabolically differentiated tissues in the body, or to different regulatory states available to the same tissue. An example of the latter would be the liver; at different times of day it either synthesizes glycogen, taking glucose from the blood, or it degrades glycogen to maintain the blood glucose level.

### HumanCyc as a tool

The HumanCyc PGDB is freely available for use by the scientific community from the SRI website [19]. Basic queries to HumanCyc can be issued through the BioCyc Query Page [20]. This page supports a number of query types. For text searches through the DB, for example, enter 'tryptophan' next to the 'Query All (by name)' box, and then click 'submit' to retrieve a list of all enzymes, pathways, compounds, and reactions whose name includes that word. Click on 'Choose from a list of pathways' to generate a list of all pathways within HumanCyc. Click 'submit' near 'Browse Ontology' to browse one of several possible classification hierarchies, such as the ontology that classifies metabolic pathways according to their physiological role.

When viewing a HumanCyc pathway display, be aware that the software omits enzyme names for pathway holes. That is, when no human protein has been identified that catalyzes a reaction within a pathway, no enzyme name is drawn next to that reaction.

The cellular overview [21], a full human metabolic pathway map, provides a tool for analysis of high-throughput datasets. The Omics Viewer [22] allows the user to visualize gene-expression data, protein-expression data, metabolomics measurements, or reaction flux data in a pathway context by painting reaction steps in the metabolic overview with colors that represent the expression levels of the genes coding for enzymes that catalyze those steps.

The PathoLogic summary page for *H. sapiens *includes a report that lists the evidence for each predicted pathway in HumanCyc, with pathways sorted according to the MetaCyc pathway ontology [23].

HumanCyc is currently available for local installation at your institution [24] as a set of flat files, and as part of an executable program running on Windows/Intel, Linux/Intel and Solaris/Sun. The latter configuration can function as both a desktop application, and as a local mirror of the HumanCyc website on the user's intranet.

### Related work

Related databases of human metabolic pathways include the GenMAPP [25] collection of 14 drawings of human metabolic pathways that are available through the web; the KEGG [26] metabolic pathway website, which allows coloring of a set of reference pathway maps to indicate which enzymatic steps have corresponding human enzymes; and the Reactome (Cold Spring Harbor Laboratory, European Bioinformatics Institute (EBI) and the Gene Ontology (GO) Consortium) project, which has curated human metabolic pathways in a web-accessible database [27].

PathoLogic was the first program for automated inference of genome-scale pathway reconstructions from genome data [28]. Additional methods for prediction of metabolic pathways include methods that infer 'extreme pathways' and 'elementary modes' from flux-balance models of reaction networks [29], which have been applied to the human mitochondrion [30]. Rather than recognizing known, historically defined pathways in a large reaction network, as does PathoLogic, these methods infer pathways from the stoichiometric matrix representing a biochemical network by convex analysis. The biological utility and meaning of these pathways, and their correspondence to known metabolic pathways that have been established through experimental studies, remains to be demonstrated. One benefit of pathway analysis by projecting previously known pathways onto a genome using PathoLogic is the fact that projection of known pathways tells us what reactions to expect to occur in another organism, leading to the identification of pathway holes, and the directed search for their fillers. In fact, the occurrence of hundreds of pathway holes in every genome we have analyzed raises the question of how the extreme pathways method can work given that the reaction networks it relies on must omit hundreds of reactions (the pathway holes) as a result of the incompleteness of genome annotations.

The following additional pathway prediction methods are related to this work, but have not been applied on a genome scale and have received little validation. Zien and colleagues use gene-expression data to score pathways that are enumerated combinatorially from a reaction database. Van Helden and colleagues use clustering of gene-expression data to generate seed reactions that are used to combinatorially enumerate pathways [31,32]. McShan *et al*. infer novel biotransformation rules for xenobiotics from the molecular graphs of compounds [33]. A recent review discusses the potential for reconstruction of metabolic networks using metabolomics technology [34].

### Limitations of HumanCyc and future work

The user of HumanCyc should be aware of several potential limitations that influence the interpretation of the DB contents. First, HumanCyc is incomplete in the sense that some known (and unknown) human metabolic pathways are not present in it. It will require a significant database curation effort to enter all known pathways. Second, HumanCyc probably contains false-positive pathway predictions. Our approach is to err on the side of being more inclusive in our pathway predictions so that all potential pathways are brought to the attention of the scientific community for evaluation. For example, HumanCyc sometimes contains multiple pathway variants that we currently lack the evidence to choose between, and in other cases the actual human pathway may be a variant of the pathway present in HumanCyc. Third, HumanCyc does not encode information about the location (compartment(s), cell type(s) or tissue(s)) in which a pathway occurs. Each pathway is defined using all identified human enzymes, meaning that location-specific versions of a pathway are not specifically identified. Furthermore, the presence of a pathway could be incorrectly inferred because the enzymes that make up a pathway are found in different locations and are never expressed in a common location, meaning that the pathway can never occur in its entirety. Fourth, HumanCyc does not contain nucleotide or amino-acid sequences. Our future work will address the first three of these limitations, and will include a significant effort to manually refine and update HumanCyc with pathway and enzyme information from the biomedical literature. To date, three pathways have been added to HumanCyc through manual curation. Experimentally elucidated pathways in HumanCyc will be annotated as such with evidence codes; pathways predicted by PathoLogic are annotated as computationally inferred.

HumanCyc can be used to develop an agenda for experimental refinement of the human metabolic map. Predicted pathways should be experimentally verified, with particular attention to choosing among multiple pathway variants. Candidate 'fillers' for the 178 unresolved pathway holes identified herein should be identified.

## Conclusions

PGDBs endow genomic information with an extended dimension that allows researchers to analyze an organism's genome with respect to the causal relationships inherent to a metabolic network. In this sense, HumanCyc provides the opportunity to look at human metabolic processes within the context of the annotated human genome, and *vice versa*. The computationally predicted metabolic network provided a rational framework for understanding the genetic basis of some well-characterized human dietary requirements, that is, 11 nonessential amino acids and 22 essential nutrients (9 essential amino-acids and 13 essential vitamins).

PathoLogic's pathway prediction process provides a reasonably accurate picture of the metabolic network, and Pathway Tools provides the user with extensive capabilities for refining the DB to reflect improvements in our understanding of the human metabolic network. The query and visualization capabilities of the Pathway Tools software (such as the visualization of gene-expression data superimposed on the metabolic map) will facilitate novel approaches for analyzing the complexity of functional relationships within the human genome.

## Materials and methods

### Data gathering and preparation

The PathoLogic program generates a new PGDB starting from the annotated genome of an organism, meaning a complete genome sequence (closed or gapped) for which gene prediction and sequence analyses have already identified the locations of likely coding regions, and have predicted the functions of these genes. We used Ensembl Build 31 as our main data source for the annotated human genome, and complemented that information with data from LocusLink, the National Center of Biotechnology Information (NCBI) database of genetic loci. We used GenBank as our source for the mitochondrion genome, as this information was not included in Ensembl. LocusLink mitochondrial loci were used to complement this information when applicable.

The information from these different sources required special preprocessing to make it available to PathoLogic. That preprocessing addressed three needs: first, to convert the disparate data formats used by these sources into a format parsable by PathoLogic; second, to extract information useful to PathoLogic; and third, to remove redundancy among the sources.

In the case of Ensembl, the standard Ensembl flat files included just a subset of the information needed for the PGDB generation process. We therefore used the EnsMart facility provided by Ensembl [5] to generate files with the required data. EnsMart allows the user to select subsets of the genome (from small regions to entire chromosomes) and output desired data about each gene in different tabular formats. Additional data file 1 lists the genetic element files generated using EnsMart. One file was generated for each human chromosome and contig, with the file names corresponding to the chromosome and contig names in Ensembl. When the corresponding chromosome for a given contig was known, the contig's file name would be constructed by prepending the chromosome's name to the contig's name, otherwise 'Un', for unknown, was prepended to the contig's name.

We selected LocusLink file LL3_030319 (Version from 19 March, 2003), which contained all data required by PathoLogic (the LocusLink organism-specific files, like the Ensembl files, did not include all the needed database fields). Table 7 shows the types of information extracted from each source when available. For example, we included a large number of comments extracted from LocusLink in HumanCyc; each such comment in HumanCyc ends with a citation to LocusLink to properly attribute its source. Function descriptions for gene products are very important for PathoLogic, as they will be matched against enzyme activity names stored in MetaCyc, a multi-organism database of experimentally determined metabolic pathways and enzymes [15]. In Ensembl, such functional information had to be parsed from the 'function' field in EnsMart data. This field sometimes includes long and complex descriptions of the gene product's function, mostly extracted by Ensembl from SwissProt [35]. These descriptions could include synonyms, EC numbers, and multiple functions (see Figure 1). All that information had to be extracted and presented to PathoLogic in a structured form.

When generating the input files for PathoLogic, information from Ensembl and LocusLink was combined only when the Ensembl record for a gene included a cross reference to a LocusLink ID. In such a case, data from the LocusLink entry was merged with that of the Ensembl gene, meaning that when both databases provide an attribute such as a gene name, the Ensembl data is preferred. This approach created gene objects for HumanCyc that include information from both the Ensembl and the LocusLink entries. Those HumanCyc gene objects use the Ensembl ID as their unique identifier, and have database links to the corresponding LocusLink entries. For the mitochondrial data, the number of LocusLink mitochondrial loci was small enough so that they were easily checked for matches with corresponding genes in the GenBank files.

LocusLink loci that had no direct counterpart in either the Ensembl data or the GenBank mitochondrial data (for example, LocusLink loci that were not directly referenced in any Ensembl gene record) were assigned their own record in the PathoLogic input files. The LocusLink ID was used as the unique identifier for these gene objects. Only loci corresponding to 'real' genes (not models, phenotypes or pseudogenes) were included in HumanCyc. We must point out that records corresponding only to LocusLink loci lack gene position information, so they cannot be precisely placed on a chromosome map. We were aware that adding these LocusLink-only-based records to HumanCyc would produce some redundancy in the database, but this was accepted for the sake of completeness. Manual analysis of similar Ensembl and LocusLink-based gene objects after building HumanCyc led to the fusion of gene objects corresponding to the same gene. The number of LocusLink gene objects that were not merged to corresponding gene objects from Ensembl or GenBank is shown in the 'nonredundant' column of Table 7.

It is readily apparent from Table 7 that HumanCyc, thanks to the combination of the Ensembl and LocusLink data sources, has excellent cross-reference coverage to many other biological databases (including Ensembl and LocusLink themselves). In addition to the databases mentioned in Table 7, we added links to the GeneCards genomic database for those genes with known HUGO IDs.

Seventy-six PathoLogic input files were generated from the preceding data sources: 24 for the human chromosomes, one for the mitochondrion, 50 for the different contigs not yet integrated to the chromosomal sequences, and one called 'unknown' for all the loci that had no chromosome information. A replicon object was created in HumanCyc for each of these files.

### Prediction of human metabolic pathways using PathoLogic

This section summarizes the PathoLogic algorithm (for a more detailed description of the method see [36,37]). For an evaluation of the accuracy of PathoLogic see [36].

After initializing the schema of the new PGDB, a database object is created for each replicon and contig, and for each gene and its corresponding gene product. PathoLogic then tries to determine the metabolic reaction catalyzed (if any) by each gene product in the organism by using its EC number, if provided in the annotation, and by matching the name of each gene product against the extensive dictionary of enzyme names within the MetaCyc DB [15]. Finally, the list of reactions now known to be catalyzed by the organism is matched against all the pathways in MetaCyc. For pathways with significant numbers of matches (see [36] for a detailed description of this algorithm), PathoLogic imports the pathway and its associated reactions and substrates from MetaCyc into the new PGDB. This method of pathway prediction is analogous to predicting the function of a protein based on sequence similarity to a protein of known function, in that both methods recognize the presence of something known (a known pathway versus a known protein function) based on a similarity between patterns (a pattern of enzymes present versus a sequence pattern). The two methods share similar limitations: just as sequence similarity cannot predict protein functions that are not in the sequence database, PathoLogic cannot predict pathways that are not in MetaCyc.

As mentioned above, PathoLogic will assign reactions for those enzymes that have an exact EC number match or name match against MetaCyc. A gene product name may not exactly match that of any enzyme in MetaCyc. Some enzyme names will produce ambiguous matches or no match at all. PathoLogic assembles a list of 'probable enzymes' that includes both ambiguous matches and nonmatched proteins whose names suggest enzymatic activity. This list is examined manually through a PathoLogic module that helps the user evaluate possible matching candidates within MetaCyc and assign probable enzymes to the correct reaction, if possible.

An alternative to our strategy of using the existing EC number and function assignments from LocusLink and Ensembl would be for us to discard those assignments and to reanalyze the genome using sequence analysis methods to produce new assignments. We rejected this approach for two reasons: first, it would discard some experimentally derived function assignments in place of less reliable computational assignments; and second, we consider the Ensembl function predictions to be of high quality, and we are aware of no evidence that our group, or any other group, has a sequence analysis methodology that will produce function assignments that are substantially more accurate than those of Ensembl.

Finally, PathoLogic generates reports summarizing the amount of evidence supporting each pathway in the new PGDB, and listing the pathway holes, that is, the enzymes missing from each predicted pathway. This information helps the user identify pathways that should be deleted from the PGDB, such as variant pathways and false-positive predictions made by PathoLogic. For example, MetaCyc includes eight variants of the TCA cycle. Several of these might appear in a newly predicted PGDB. False-positive pathways, some of which are predicted because they share reactions with other pathways in MetaCyc, should be removed from the PGDB. Once variant or false-positive pathways are eliminated by the user, PGDB generation has been completed.

### Filling holes in HumanCyc pathways

To determine the function of a protein sequence, researchers typically use a single sequence to search for potential homologs in a large public database. To identify sequences to fill pathway holes, we have, in effect, reversed this search process. We search the genome for a sequence that will provide the enzymatic function needed to fill each pathway hole. Our method uses multiple isozyme sequences (retrieved via MetaCyc from Swiss-Prot and PIR) to search a genome for similar sequences (hole-filler candidates). We then evaluate each candidate to determine the probability that the sequence has the desired function based on homology and pathway-based data. A hole-filler tool that implements this pathway-driven gene-finding methodology has been developed [9].

### Analysis of HumanCyc metabolic network

Once HumanCyc was built and manually refined, as explained above, we examined the metabolic network within HumanCyc in order to make a preliminary assessment of the quality of PathoLogic's predictions and to check for pathways not previously thought to occur in humans. We also compared the metabolic network of HumanCyc to that of two of our curated PGDBs, corresponding to a bacterium and a plant. These PGDBs are EcoCyc (*E. coli*) and AraCyc (*A. thaliana*).

## Additional data files

The following additional data are available with the online version of this article. Additional data file 1 contains a table listing the data file names generated from EnsMart for each human chromosome or contig and provided as input to PathoLogic, thus indicating which contigs are associated with which chromosomes.

## Supplementary Material

Additional data file 1 A table listing the data file names generated from EnsMart for each human chromosome or contig and provided as input to PathoLogic, thus indicating which contigs are associated with which chromosomes Click here for additional data file

## Figures and Tables

**Table 1:** The number of human proteins that were assigned enzyme activities (which caused them to become connected to reaction objects within HumanCyc), according to the mechanism of reaction matching

Type of match	Number of proteins
PathoLogic matched by EC number	2,057
PathoLogic matched by name	314
Ambiguous	27
Unmatched by PathoLogic	27,185
Probable enzymes	1,320
Manually matched	625

**Table 2:** HumanCyc statistics

PGDB objects	Quantity
Replicons	76
Genes	28,783
Protein genes	28,583
Enzyme genes	2,742
RNA genes	200
	
tRNAs	50
Compounds	661
Polypeptides	28,602
Protein complexes	22
Enzymes	2,709
	
Enzymatic Reactions	1,093
With enzyme in HumanCyc	896
Pathways	135
Database links	389,262
Citations	41,810

**Table 3:** The entire set of pathways in HumanCyc, grouped by classes using the MetaCyc pathway classification hierarchy

Class	Subclass	Pathway	EcoCyc	AraCyc
Biosynthesis	Polyamines	Betaine biosynthesis	*	*
		Betaine biosynthesis II		
		Spermine biosynthesis		*
		Polyamine biosynthesis II		
		Ornithine spermine biosynthesis		*
		Polyamine biosynthesis	*	*
		UDP-*N*-acetylgalactosamine biosynthesis		*
		UDP-*N*-acetylglucosamine biosynthesis	*	
	Nucleotides	*De novo *biosynthesis of purine nucleotides	*	
		Purine and pyrimidine metabolism		
		Purine biosynthesis 2		
		*De novo *biosynthesis of pyrimidine ribonucleotides	*	
		Salvage pathways of pyrimidine ribonucleotides	*	
		*De novo *biosynthesis of pyrimidine deoxyribonucleotides	*	
		Salvage pathways of pyrimidine deoxyribonucleotides	*	
	Fatty acids and lipids	Fatty acid elongation - saturated	*	*
		Fatty acid biosynthesis - initial steps	*	*
		Phospholipid biosynthesis	*	*
		Phospholipid biosynthesis II		
		Mevalonate pathway		*
		Triacylglycerol biosynthesis		*
	Cofactors, prosthetic groups, electron carriers	Heme biosynthesis II		
		NAD biosynthesis II		
		NAD biosynthesis III		
		NAD phosphorylation and dephosphorylation	*	
		Pyridine nucleotide biosynthesis	*	*
		Pyridine nucleotide cycling	*	
		Glutathione-glutaredoxin redox reactions	*	
		Glutathione biosynthesis	*	*
		Thioredoxin pathway	*	*
		Pantothenate and coenzyme A biosynthesis	*	*
		Pyridoxal 5'-phosphate salvage pathway	*	*
		FormylTHF biosynthesis	*	*
		Polyisoprenoid biosynthesis	*	*
		Methyl-donor molecule biosynthesis	*	
	Cell structures	Colanic acid building blocks biosynthesis	*	*
		GDP-mannose metabolism	*	*
		Mannosyl-chito-dolichol biosynthesis		*
		UDP-*N*-acetylglucosamine biosynthesis	*	
	Carbohydrates	GDP-D-rhamnose biosynthesis		
		Gluconeogenesis	*	*
		Mannosyl-chito-dolichol biosynthesis		*
		Trehalose degradation - low osmolarity	*	*
	Aminoacyl-tRNAs	tRNA charging pathway	*	*
	Amino acid biosynthesis	Alanine biosynthesis II		*
		Arginine biosynthesis 4		*
		Citrulline biosynthesis		
		Asparagine biosynthesis I		
		Aspartate biosynthesis II		
		Cysteine biosynthesis II		
		Glutamate biosynthesis II		*
		Glutamine biosynthesis II		
		Glycine cleavage	*	
		Glycine biosynthesis I	*	*
		Methionine salvage pathway		
		Proline biosynthesis I	*	*
		Serine biosynthesis	*	*
		Tyrosine biosynthesis II		
				
Degradation	Sugars and polysaccharides	Lactose degradation 4		*
		Lactose degradation 2		*
		Sucrose degradation III		
		Galactose metabolism	*	*
		Glucose 1-phosphate metabolism	*	*
		Glycogen degradation	*	*
		Mannose degradation	*	
		Non-phosphorylated glucose degradation		*
		UDP-glucose conversion		*
		Ribose degradation	*	*
		Trehalose degradation - low osmolarity	*	*
	Sugar derivatives	Lactate oxidation		
		Mannitol degradation	*	
		Sorbitol degradation	*	
		Glucosamine catabolism	*	
	Other degradation	Removal of superoxide radicals	*	*
		Methylglyoxal degradation		
	Nucleosides and nucleotides	(Deoxy)ribose phosphate metabolism	*	*
		Periplasmic NAD degradation		
	Fatty acids	Fatty acid oxidation pathway	*	*
		Triacylglycerol degradation		*
		Lipases pathway		*
	Carboxylates, other	Propionate metabolism - methylmalonyl pathway	*	
		2-Oxobutyrate degradation		
		Acetate degradation	*	*
		Pyruvate metabolism		
		*N*-acetylneuraminate degradation		
	C1 compounds	Carbon monoxide dehydrogenase pathway		*
		Serine-isocitrate lyase pathway		*
	Amino acids, amines	Alanine degradation 3		*
		Arginine degradation III		
		Arginase degradation pathway		
		Arginine proline degradation		*
		Asparagine degradation 1		*
		Aspartate degradation 1		
		Malate/aspartate shuttle pathway		
		L-cysteine degradation IV		*
		L-cysteine degradation VI		
		Cysteine degradation I		
		Glutamate degradation I		*
		Glutamate degradation IV		
		Glutamate degradation VII		*
		Glutamine degradation 1		
		Glutamine degradation II		
		Glycine degradation II		
		Glycine degradation I		
		Histidine degradation III		
		Histidine degradation I		
		Homocysteine degradation I		
		Isoleucine degradation I		*
		Isoleucine degradation III		
		Leucine degradation II		
		Leucine degradation I		*
		Lysine degradation I		*
		Methionine degradation 1		*
		4-Hydroxyproline degradation		*
		*S*-adenosylhomocysteine degradation		
		Phenylalanine degradation I		
		Proline degradation III		
		Proline degradation II		
		L-serine degradation	*	*
		Threonine degradation 2		
		Tryptophan degradation I		
		Tryptophan degradation III		*
		Tryptophan kynurenine degradation		
		Tyrosine degradation		
		Valine degradation I		*
	Alcohols	Aerobic glycerol degradation II		*
		Glycerol metabolism	*	*
		Glycerol degradation I	*	
		Ethanol degradation	*	
	Amines and polyamines, other	Citrulline degradation		
		*N*-acetylglucosamine, *N*-acetylmannosamine and *N*-acetylneuraminic acid dissimilation	*	*
		Glucosamine catabolism	*	
				
Energy metabolism		Glycolysis 3		*
		Glycolysis	*	*
		Glycolysis 2		
		Glyceraldehyde 3-phosphate degradation		*
		Non-oxidative branch of the pentose phosphate pathway	*	*
		Oxidative branch of the pentose phosphate pathway	*	*
		Aerobic respiration - electron donors reaction list	*	
		Pyruvate dehydrogenase	*	*
		TCA cycle - aerobic respiration	*	*
		Entner-Doudoroff pathway	*	

More detailed subclasses were not included for brevity. An asterisk in one of the last two columns means that the pathway is also present in the EcoCyc (*E. coli*) and/or AraCyc (*A. thaliana*) databases, respectively. Note that pathway names are derived from the MetaCyc database, which explains why HumanCyc contains a pathway called 'Heme Biosynthesis II' but not 'Heme Biosynthesis I.'

**Table 4:** A comparison of candidates for three missing enzymes

Candidate		P (has-function)	Number of hits	Best E-value	Average rank	Percentage of query aligned
Reaction hole: imidazolonepropionase						
A	ENSG00000139344-MONOMER Functional annotation: UNKNOWN	0.98	28	7.0e-69	1.0	91.9
B	ENSG00000119125-MONOMER Functional annotation: Guanine deaminase	0.00018	6	3.0e-6	3.5	37.9
						
Reaction hole: *N*-acetylglucosamine-6-phosphate deacetylase						
C	ENSG00000162066-MONOMER Functional annotation:CGI-14 protein	0.998	9	1e-110	1.0	94.6
D	ENSG00000119125-MONOMER Functional annotation: Guanine deaminase	1.0e-5	4	0.85	4.0	19.9
						
Reaction hole: aldose 1-epimerase						
E	ENSG00000143891-MONOMER Functional annotation:AMBIGUOUS	0.98	19	3e-74	1.58	81.9
F	ENSG00000117308-MONOMER Functional annotation:UDP-glucose 4-epimerase	0.93	4	1e-100	1.0	58.3

**Table 5:** Comparison of known essential human nutrients with corresponding biosynthetic pathways in MetaCyc and in HumanCyc

Essential nutrient in humans	Biosynthetic pathway in MetaCyc?	Biosynthetic pathway inferred in humans?
Amino acids		
Arginine	Y	N
Histidine	Y	N
Isoleucine	Y	N
Leucine	Y	N
Lysine	Y	N
Methionine	Y	N
Phenylalanine	Y	N
Threonine	Y	N
Valine	Y	N
		
Vitamins		
Ascorbic acid (Vitamin C)	Y	N
Biotin (Vitamin H)	Y	N
Folic acid (Vitamin M)	Y	N
Niacin (Vitamin B_3_)	N	N
Pantothenic acid	Y	Y
Pyridoxine (Vitamin B_6_)	N	N
Riboflavin (Vitamin B_2_)	Y	N
Thiamine (Vitamin B_1_)	Y	N
Cobalamin (Vitamin B_12_)	Y	N
Retinol (Vitamin A)	N	N
Vitamin D	N	N
Tocopherol (Vitamin E)	N	N
Vitamin K	N	N

Note that a pathway cannot be predicted in HumanCyc if it does not exist in MetaCyc.

**Table 6:** Pathways (including superpathways) that are common to human, bacteria and plant PGDBs

Class	Subclass	Pathway
Biosynthesis	Polyamines	Betaine biosynthesis
		Polyamine biosynthesis
	Fatty acids and lipids	Phospholipid biosynthesis
		Fatty acid biosynthesis - initial steps
		Fatty acid elongation - saturated
	Cofactors, prosthetic groups, electron carriers	Pyridine nucleotide biosynthesis
		Thioredoxin pathway
		Glutathione biosynthesis
		Pantothenate and coenzyme A biosynthesis
		Pyridoxal 5'-phosphate salvage pathway
		Polyisoprenoid biosynthesis
		FormylTHF biosynthesis
	Cell structures	Colanic acid building blocks biosynthesis
		GDP-mannose metabolism
	Carbohydrates	Gluconeogenesis
		Trehalose degradation - low osmolarity
	Aminoacyl-tRNAs	tRNA charging pathway
	Amino acid biosynthesis	Proline biosynthesis I
		Glycine biosynthesis I
		Serine biosynthesis
		
Degradation	Sugars and polysaccharides	Glucose 1-phosphate metabolism
		Galactose metabolism
		Trehalose degradation - low osmolarity
		Glycogen degradation
		Ribose degradation
	Other degradation	Removal of superoxide radicals
	Nucleosides and nucleotides	(Deoxy)ribose phosphate metabolism
	Fatty acids	Fatty acid oxidation pathway
	Carboxylates, other	Acetate degradation
	Amino acids, amines	L-serine degradation
	Alcohols	Glycerol metabolism
		
Energy metabolism		Pyruvate dehydrogenase
		TCA cycle - aerobic respiration
		Glycolysis
		Oxidative branch of the pentose phosphate pathway
		Nonoxidative branch of the pentose phosphate pathway

HumanCyc, *H. sapiens*; EcoCyc, *E. coli*; AraCyc, *A. thaliana*. The pathways in the table are included in all three PGDBs.

**Table 7:** Information extracted from different data sources

Data source (version)	Information extracted (for each gene or locus)	Number of genes	
		
		Obtained	Nonredundant
Ensembl (Build 31)	Gene name, chromosome or contig, start and end positions, strand (transcription direction), exons, gene-product (including function name(s) or description(s), synonyms and EC number(s)), cross references (IDs) to other databases (SwissProt, HUGO, PDB, GO, RefSeq, OMIM, Entrez, SPTREMBL, EMBL, LocusLink).	24,847	
LocusLink (03/29/2003)	Gene name, chromosome, gene product (function name or description), function synonyms, EC number(s), gene and protein comments, cross references (IDs) to other databases (Entrez, UCSC Genome, RefSeq, GO, OMIM, UniGene, PubMed)	18,880	3,936
GenBank NC_001807 (mitochondrion)	Gene name, start and end positions, transcription direction, gene product (function name or description)	35	

Functional information in Ensembl had to be extensively parsed to extract multiple functions, EC numbers, and/or synonyms. The 'nonredundant' column shows the number of genes from LocusLink that had no corresponding gene in the other two data sources (Ensembl and GenBank).
