# Supplementary material for: Computational prediction of human metabolic pathways from the complete human genome
Source: Genome Biol. 2004 Dec 22;6(1):R2. doi: 10.1186/gb-2004-6-1-r2 (PMC549063; doi:10.1186/gb-2004-6-1-r2)
Supplement: Additional data file 1 — A table listing the data file names generated from EnsMart for each human chromosome or contig and provided as input to PathoLogic, thus indicating which contigs are associated with which chromosomes [file gb-2004-6-1-r2-s1.html]

HumanCyc Genomic Elements


|  |  |
| --- | --- |
| Chromosome Files | Contig Files |
| 1 | 1\_NT\_037502, 1\_NT\_037506 |
| 2 | 2\_NT\_034507 |
| 3 | 3\_NT\_037563, 3\_NT\_037576 |
| 4 | 4\_NT\_034730, 4\_NT\_034738, 4\_NT\_034748, 4\_NT\_034756 |
| 5 |  |
| 6 | 6\_DR51, 6\_DR52 |
| 7 |  |
| 8 | 8\_NT\_037731 |
| 9 | 9\_NT\_035019 |
| 10 | 10\_NT\_035057 |
| 11 | 11\_NT\_024164, 11\_NT\_024225, 11\_NT\_035130, 11\_NT\_035133, 11\_NT\_037781 |
| 12 | 12\_NT\_030816, 12\_NT\_035266, 12\_NT\_035283, 12\_NT\_037813, 12\_NT\_037818, 12\_NT\_037834 |
| 13 |  |
| 14 | 14\_NT\_037848 |
| 15 | 15\_NT\_035330, 15\_NT\_035341, 15\_NT\_037874 |
| 16 | 16\_NT\_035396, 16\_NT\_037910, 16\_NT\_037915 |
| 17 | 17\_NT\_010845, 17\_NT\_024898, 17\_NT\_030164, 17\_NT\_035470, 17\_NT\_035479, 17\_NT\_037974, 17\_NT\_037975, 17\_NT\_037978 |
| 18 |  |
| 19 | 19\_NT\_035597, 19\_NT\_035607 |
| 20 |  |
| 21 |  |
| 22 |  |
| X |  |
| Y |  |
| Unknown | Un\_NT\_035694, Un\_NT\_035721, Un\_NT\_035744, Un\_NT\_035745, Un\_NT\_038071, Un\_NT\_038089, Un\_NT\_038090, Un\_NT\_038115 |
